# Alleviation of Water Stress Effects on MR220 Rice by Application of Periodical Water Stress and Potassium Fertilization 

**DOI:** 10.3390/molecules19021795

**Published:** 2014-02-05

**Authors:** Nurul Amalina Mohd Zain, Mohd Razi Ismail, Maziah Mahmood, Adam Puteh, Mohd Hafiz Ibrahim

**Affiliations:** 1 Institute of Tropical Agriculture, Universiti Putra Malaysia, 43400 UPM Serdang, Selangor Darul Ehsan, Malaysia; E-Mail: jae.amalina@gmail.com; 2 Institute of Biological Science, Faculty of Science, University Malaya, 50603, Kuala Lumpur, Malaysia; 3 Department of Biochemistry, Faculty of Bioctechnology and Biomolecular Science, Universiti Putra Malaysia, 43400 UPM Serdang, Selangor Darul Ehsan, Malaysia; E-Mail: maziahm@upm.edu.my; 4 Department of Crop Science, Faculty of Agriculture Science, Universiti Putra Malaysia, 43400 UPM Serdang, Selangor Darul Ehsan, Malaysia; E-Mail: adamputeh@upm.edu.my; 5 Department of Biology, Faculty of Science, Universiti Putra Malaysia, 43400 UPM Serdang, Selangor Darul Ehsan, Malaysia; E-Mail: mhafizphd@yahoo.com

**Keywords:** water stress, rice production, periodical water stress, potassium fertilization, leaf gas exchanges, biochemical changes, chlorophyll fluorescence

## Abstract

The use of periodical water stress and potassium fertilization may enhance rice tolerance to drought stress and improve the crop’s instantaneous water use efficiency without much yield reduction. This study was conducted to assess the effects of different periodical water stress combined with potassium fertilization regimes on growth, yield, leaf gas exchanges and biochemical changes in rice grown in pots and compare them with standard local rice grower practices. Five treatments including (1) standard local grower’s practice (control, 80CF = 80 kg K_2_O/ha + control flooding); (2) 120PW15 = 120 kg K_2_O/ha + periodical water stress for 15 days; (3) 120DS15V = 120 kg K_2_O/ha + drought stress for 15 days during the vegetative stage; (4) 120DS25V = 120 kg K_2_O/ha + drought stress for 25 days and (5) 120DS15R = 120 kg K_2_O/ha + drought stress for 15 days during the reproductive stage, were evaluated in this experiment. Control and 120PW15 treatments were stopped at 100 DAS, and continuously saturated conditions were applied until harvest. It was found that rice under 120PW15 treatment showed tolerance to drought stress evidenced by increased water use efficiency, peroxidase (POX), catalase (CAT) and proline levels, maximum efficiency of photosystem II (f_v_/f_m_) and lower minimal fluorescence (f_o_), compared to other treatments. Path coefficient analysis revealed that most of parameters contribute directly rather than indirectly to rice yield. In this experiment, there were four factors that are directly involved with rice yield: grain soluble sugar, photosynthesis, water use efficiency and total chlorophyll content. The residual factors affecting rice yield are observed to be quite low in the experiment (0.350), confirming that rice yield was mostly influenced by the parameters measured during the study.

## 1. Introduction

Drought is an abiotic stress, and it affects plants at various levels of their organization. Under prolonged drought, many plants will dehydrate and die. Water stress in plants reduces the plant-cell’s water potential and turgor, which elevate solute concentrations in the cytosol and extracellular matrices. As a result, cell enlargement decreases leading to growth inhibition and reproductive failure [1], which is followed by accumulation of abscisic acid (ABA) and compatible osmolytes like proline, which cause wilting. Drought not only affects plant-water relations through the reduction of water content, turgor and total water, but it also affects stomatal closure, limits gaseous exchange, reduces transpiration and arrests carbon assimilation (photosynthesis) rates [2]. Negative effects on mineral nutrition (uptake and transport of nutrients) and metabolism leads to a decrease in the leaf area and alteration in assimilate partitioning among the organs. Plant responses to drought are complex and several different mechanisms are adopted by plants when they encounter drought [3,4], including: (i) drought escape by rapid development which allows plants to finish their cycle before severe water stress; (ii) drought avoidance by, for instance, increasing water uptake and reducing transpiration rate by the reduction of stomatal conductance and leaf area; (iii) drought tolerance by maintaining tissue turgor during water stress via osmotic adjustment which allows plants to maintain growth under water stress, and (iv) resisting severe stress through other survival mechanisms [3].

Rice is one of the crops that are exposed to many environmental stresses. Lack of adequate water leading to drought stress is common in upland cultivation systems. On an average, rice needs 5,000 L of water to produce one kilogram of grain [5]. More than half of the 40 million hectares of rain-fed lowland rice worldwide suffer water scarcity at some growth stage [6]. Drought stress reduces the rice growth, and severely affects the seedling biomass, photosynthesis, stomatal conductance, plant water relations and starch metabolism [7]. Depending on timing, duration and severity of the plant water deficit, the grain yield of some rice genotypes could be reduced by up to 81% under drought [8].

The application of periodical water stress and potassium fertilization has been reported to induce tolerance of rice to osmotic stress [2]. The maintenance of high plant water status and plant functions at low plant water potential, and the recovery of plant function after water stress are the major physiological processes that contribute to the maintenance of high yield under cyclic drought period conditions [9,10,11,12,13]. In water stressed plants, increased abscisic acid (ABA) levels are known to stimulate the release of potassium from guard cells, giving rise to stomatal closure [14]. Numerous studies have shown that the application of K fertilizer mitigates the adverse effects of drought on plant growth [15,16]. Potassium increases the plant’s drought resistance through its functions in stomatal regulation, osmoregulation, energy status, charge balance, protein synthesis, and homeostasis [17]. In plants coping with drought stress, the accumulation of K^+^ may be more important than the production of organic solutes during the initial adjustment phase, because osmotic adjustment through ion uptake like K^+^ is more energy efficient [18]. Li [19] has reported that lower water loss in plants well supplied with K^+^ is due to a reduction in transpiration which not only depends on the osmotic potential of mesophyll cells, but also is controlled to a large extent by opening and closing of stomata.

Drought stress in plant cells leads to a reduction in carbon assimilation, which is linked to a physiological closure of leaf stomata and to biochemically determined lower photosynthetic activity, which affects carbohydrate economy [20]. Soluble sugars act as osmolytes maintaining cell turgor of leaves, protecting the integrity of the membrane, and preventing the denaturation of proteins [21]. Sucrose plays an important role in plant metabolism at both cellular and whole organism level. It participates not only in the response to abiotic stresses, but also serves as a nutrient and signaling molecule, modulating a wide range of gene activity [22].

Proline accumulation plays a highly protective role in plants that are exposed to abiotic stresses, being involved in osmotic adjustment together with an increase in the levels of other osmolytes [23,24]. Proline has also been suggested to act as antioxidant and to interact with hydrophobic residues in proteins [25,26]. Moreover, at high concentrations, it has little or no perturbing effect on macromolecule-solvent interactions. It was also shown that proline did not impact cell membrane functioning or antioxidant enzyme (APX, CAT, SOD) activity and contributed to mitigating the negative impact of dehydration [27]. Proline is associated with adaptation to stress, causing the expression of genes which protect cells against dehydration. Previous studies have shown that the application of periodical water stress and potassium fertilization can mitigate water stress effects on rice. However, there were no documentation on growth, leaf gas exchange and phytochemical responses. This information is important, and will be useful in rice cultivation under limiting water resources. It was hypothesized that the use of periodical water stress and potassium fertilization with 120 kg K_2_O/ha for 15 days (120PW15) could mitigate the water stress effects by increasing rice tolerance to water stress by having high production of antioxidative enzymes, proline and lipid peroxidation. The aim of the present work was to evaluate the effect of periodical water stress and potassium fertilization regimes on drought stress tolerance-related parameters (growth and yield, leaf gas exchange, chlorophyll fluorescence, the anti-oxidative enzymes (CAT and POX), soluble sugars, proline, and lipid peroxidation responses). The proposed treatment was compared with following treatments: (1) standard local grower’s practice (control, 80CF = 80 kg K_2_O/ha + control flooding); (2) 120DS15V = 120 kg K_2_O/ha + drought stress for 15 days during the vegetative stage, (3) 120DS25V = 120 kg K_2_O/ha + drought stress for 25 days during the vegetative stage, and (4) 120DS15R = 120 kg K_2_O/ha + drought stress for 15 days during the reproductive stage. The second objective was to characterize rice tolerance under 120PW15 and lastly to identify which parameters are directly and indirectly involved with rice yield under the experimental conditions by using path coefficient analysis.

## 2. Results and Discussion

### 2.1. Growth and Yield Component

The results indicated that the tested management practices influenced the yield and all the growth components (*p* ≤ 0.01; Table 1). It was observed that yield was highest under the control condition (80CF) which recorded 8.65 tonnes/ha, followed by 120DS25V (8.18 tonnes/ha), 120PW15 (7.25 tonnes/ha), and 120DS15V (7.00 tonnes/ha), while the lowest was for 120DS15R which only recorded 6.09 tonnes/ha. Panicle dry weight was found to be highest under the control treatment (126.87 kg) and lowest in the 120PW15 treatment, which only registered 99.45 kg. Application of 120 kg K_2_O/ha during stress at the vegetative stage produced highest 1,000 grain weight (26.06 g) compared to application of 120 kg K_2_O/ha at the reproductive stage during stress, that only registered 23.73 g. The root weights were 25, 29, 35 and 36% lower in the 120DS25V, 120DS15V, 120DS15R, and 80CF treatments, respectively, compared to the 120PW15 treatment, which recorded 79.97 g. There was no significant difference between 120PW15 and 120DS25V which recorded the highest panicle numbers (no m^−2^) of 326.30 and 324.68, respectively, followed by 80CF (313.31), 120DS15V (293.51) and 120DS15R (279.2). The difference in the yield between the control (80CF) and 120DS25V was just 5.5%, which showed that rice can tolerate drought stress with minimum effects on yield for 25 days if 120 kg K_2_O/ha was applied at the vegetative stage. Under prolonged drought stress for 25 days rice plants will reduce the time to flowering and thus enhance the time for grain filling.The application of 120PW15 enhanced rice tolerance to water stress and resulted in the third highest yield of 7.25 tonnes/ha. The lowest yield was recorded in 120DS15R which showed a 30% reduction in rice yield compared to the control. The present data indicated that application of potassium at 120 kg K_2_O/ha can mitigate up to 25 days of drought stress during the vegetative stage. If drought stress was initiated during the reproductive stage yields were significantly reduced. Thus, the reproductive stage is a very sensitive period. The implications of water stress during this stage are that it would reduce the rice yield, characterized by a reduction in panicle no m^−2^, root dry weight and 1,000 grain weight. The growth stimulation of rice with potassium fertilization under abiotic stress was also observed by other researchers [28,29]. This suggests the importance of potassium as a growth promoter under water stress conditions. The significant increase in root dry weight in 120PW15 compared to the other treatments might be due to the long duration of the water stress exposure compared to other treatments that only induced (water stress) during the vegetative and reproductive stages [30,31]. Application of potassium at 120 kg/ha has been shown to enhance the root dry weight during this cylic water stress period. According to Wang *et al.* [32] adequate potassium application during water stress would enhance plant drought tolerance by increasing root growth to increase the root surface and elongation that was exposed to soil to enhance root water uptake [33,34]. It was observed that rice yield had a significant positive correlation with grain sugar (r^2^ = 0.812; *p* ≤ 0.05; Table 2). This showed that higher yields are dependent on accumulation of soluble sugars during grain filling, and higher sink strength characterized by high soluble sugars in the grain might enhance rice yields [35]. Although the rice yield difference between the control and 120DS25V was only 5.5% compared to 120PW15 which was 16%, the application of periodical water stress for 15 days with 120 kg K_2_O/ha was the best practice as it was both water efficient and built tolerance of rice to drought stress. This can be recommended as a good practice when facing unexpected drought seasons.

**Table 1:** Impact of potassium fertilization on growth and yield characteristics of rice under water stress at different growth stages. Data are means ± standard error of difference between means (SEM). N = 12. Bars represent standard error of differences between means. Means not sharing a common single letter were significantly different at *p* ≤ 0.05.

Treatment	Panicle dry weight (g)	1000 grains weight (g)	Root dry weight (g)	Panicle no/m^2^	Yield (tones/ha)
80CF	126.87 ± 7.61 ^a^	25.50 ± 0.23 ^b^	49.94 ± 0.99 ^e^	313.31 ± 8.23 ^b^	8.65 ± 0.32 ^a^
120PW15	99.45 ± 5.51 ^e^	24.55 ± 0.22 ^c^	79.97 ± 2.11 ^a^	326.30 ± 5.44 ^a^	7.25 ± 0.31 ^c^
120DS15V	117.11 ± 3.42 ^c^	25.38 ± 0.23 ^b^	56.51 ± 2.32 ^c^	293.51 ± 3.45 ^c^	7.00 ± 0.32 ^d^
120DS25V	120.38 ± 4.34 ^b^	26.06 ± 0.21 ^a^	59.13 ± 2.12 ^b^	324.68 ± 3.42 ^a^	8.18 ± 0.42 ^b^
120DS15R	103.30 ± 4.55 ^d^	23.73 ± 0.24 ^d^	51.26 ± 1.23 ^d^	279.22 ± 1.24 ^d^	6.09 ± 0.41 ^e^

*Notes*: 80CF = continuous flooding + 80 kgK_2_O/ha; 120CW15 = periodical water stress for 15 days + 120 kg K_2_O/ha; 120DS15V = drought stress for 15 days at the vegetative stage + 120 kg K_2_O/ha; 120DS25V = drought stress for 25 days at the vegetative stage + 120 kg K_2_O/ha; 120DS15R = drought stress 15 days at the reproductive stage + 120 kg K_2_O/ha.

Path coefficient analysis provides an insight into the inter-relationship of various characteristics with rice grain yield. Considering the grain yield as the artifact of all the causal characters (growth, biochemical and leaf gas exchanges), the correlation coefficients of these causal factors with rice yield are partitioned into direct and indirect effects. Table 3 deals with the partitioning of correlation coefficients into direct and indirect effects of growth factors in relation to rice yield. Among the component characteristics, panicle dry weight showed highest direct positive effects (0.5671) on rice yield, along with other indirect effects. The panicle/m^2^ grain weight showed the second strongest direct effect (0.2876), followed by 1,000 grain weight (0.2111) and root dry weight (0.1001). The indirect effects showed lower correlation coefficients than direct effects, thus indicate that direct effects, especially panicle dry weight, influence the rice yield. Although rice yield was higher in 120DS25V compared to 120PW15 (8.18 *vs.* 7.25 tonnes/ha) the use of 120PW15 is more water efficient than 120DS25V, thus suggesting 120PW15 was more recommendable.

**Table 2:** Pearson’s correlation coefficients between all parameters.

Parameters	1	2	3	4	5	6	7	8	9	10	11	12	13	14	15	16	17	18	19
1. PDW	1.000																		
2. 1000gw	0.451	1.000																	
3. RDW	0.234	0.211	1.000																
4. P/m^2^	0.234	0.311	0.123	1.000															
5. Yield	0.567	0.234	0.124	0.321	1.000														
6. POX	0.021	0.098	0.021	0.113	0.765 **	1.000													
7.CAT	0.032	0.124	0.123	0.126	0.776 *	0.875 *	1.000												
8. Grain S	0.721 *	0.214	0.091	0.798 *	0.871 *	0.765 *	0.912 *	1.000											
9. Straw S	0.567	0.158	0.124	0.114	0.652 *	0.113	0.123	0.113	1.000										
9. MDA	0.123	0.113	0.234	0.065	0.654 *	0.665 *	0.423	0.897 *	0.123	1.000									
10. Proline	0.124	0.009	0.098	0.127	0.234	0.876 *	0.876 *	0.876 *	0.812 *	0.112 *	1.000								
11. A	0.021	0.123	0.124	0.145	0.876 *	0.867 *	0.213	0.923 *	0.721	0.123	0.098	1.000							
12. gs	0.123	0.124	0.214	0.098	0.776 *	0.123	0.021	0.776 *	0.123	0.114	0.098	0.012	1.000						
13. E	0.091	0.112	0.113	0.123	0.654 *	0.116	0.032	0.654 *	0.091	0.112	0.124	0.123	0.134	1.000					
14. WUE	0.014	0.081	0.123	0.167	0.886 *	0.113	0.021	0.553	0.734 *	0.881 *	0.214	0.213	0.091	0.234	1.000				
15. f_o_	0.123	0.213	0.114	0.245	−0.456	0.156	0.123	0.123	0.098	0.243	0.113	0.114	0.112	0.021	0.211	1.000			
16. f_v_/f_o_	0.211	0.113	0.098	0.121	0.098	0.158	0.124	0.114	0.112	0.113	0.123	0.245	0.113	0.098	0.091	0.234	1.000		
17. f_v_/f_m_	0.321	0.081	0.123	0.167	0.765 *	0.123	0.123	0.098	0.081	0.009	0.123	0.123	0.123	0.021	0.112	0.098	0.214	1.000	
18. TCC	0.125	0.123	0.224	0.132	0.876 *	0.115	0.003	0.123	0.213	0.001	0.116	0.167	0.154	0.032	0.113	0.032	0.012	0.233	1.000

*Note*: * and ** significant at *p* ≤ 0.05 and *p* ≤ 0.01 respectively. PDW = panicle dry weight; 1,000gw = 1,000 grain weight; RDW = root dry weight; P/m^2^ = panicle/m^2^; yield = rice yield; POX = peroxidase; CAT = catalase; Grain S = grain soluble sugar; straw S = straw soluble sugar; MDA = malondialdehyde; A = net photosynthesis; gs = stomatal conductance; E = transpiration rate; WUE = water use efficiency; f_o_ = initial fluorescence; f_v_/f_o_ = maximum quantum yield of photochemical and non photochemical process in photosystem II; f_v_/f_m_ = maximum efficiency of photosystem II and TCC = total chlorophyll content.

**Table 3:** Path Coefficient Analysis showing direct and indirect effects of growth characteristics with rice yield.

Characteristics	Correlation with rice yield	Direct effect	Indirect effect via			
			PDW	1000gw	RDW	P/m^2^
PDW	0.5671	0.5771	-	0.0012	0.0231	0.0321
1000gw	0.2342	0.2111	0.0021	-		0.0041
RDW	0.1242	0.1001	0.0033	0.0054	-	0.0017
P/m^2^	0.3211	0.2876	0.0123	0.0065	0.0024	-

*Note*: PDW= panicle dry weight; 1,000 gw = 1,000 grain weight; RDW = root dry weight; P/m^2^ = panicle/m^2^. Residual factor = 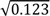
= 0.350.

### 2.2. Peroxidase Activity and Catalase Activity

**Figure 1:**
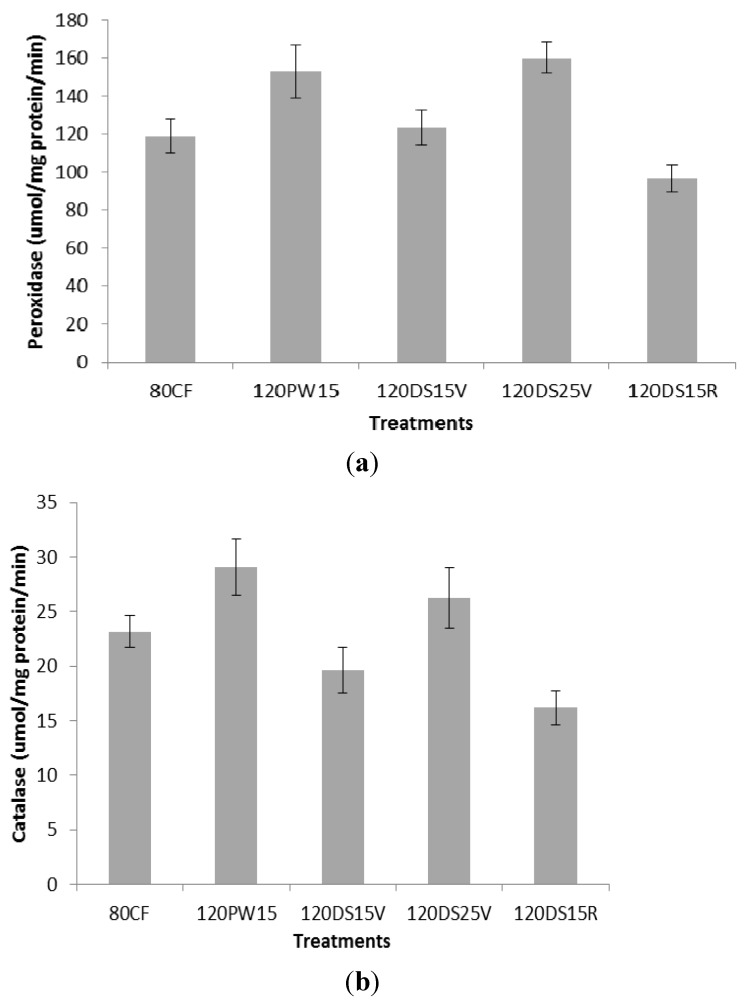
Impact of potassium fertilization on peroxidase activity (**a**) and catalase activity (**b**) under water stress treatments at different growth stages of rice N = 12. Bars represent standard error of differences between means.

*Notes*: 80CF = continuous flooding + 80 kgK_2_O/ha; 120PW15 = periodical water stress for 15 days + 120 kg K_2_O/ha; 120DS15V = drought stress for 15 days at the vegetative stage + 120 kg K_2_O/ha; 120DS25V = drought stress for 25 days at the vegetative stage + 120 kg K_2_O/ha; 120DS15R = drought stress for 15 days at the reproductive stage + 120 kg K_2_O/ha.

The treatments had a significant effect on peroxidase activity (*p* ≤ 0.05; Figure 1a). Peroxidase activity was found to be highest (159.21 µmol/mg/min) in 120DS25V, followed by 120PW15 (147.28 µmol/mg/min), 120DS15V (122.17 µmol/mg/min) and the control (118.21 µmol/mg/min), and was lowest in 120DS15R (97.28 µmol/mg/min). Similar trends was observed in catalase activity (Figure 1b). The present results indicate that application of cyclic water stress had increased the rice plants’ resistance to water stress by increasing antioxidant enyzme levels. Exposure to drought stress from 15 to 25 days at the vegetative stage increased the peroxidase and catalase activity, which were highest in the 120PW15 and 120DS25V treatments. The data indicated that the defense mechanism was highest when potassium was applied during the vegetative stage until 25 days of drought stress (120DS25V) with the provision of cyclic water stress for 15 days (120PW15) and that the defense mechanism was very important to achieve high yields during abiotic stress [36]. Under stress potassium plays an important role in the synthesis of proteins by participating in polypeptide synthesis in ribosomes, which requires a high concentration of potassium [37]. Tripathi *et al*. [38] reported that proteins such as thioredoxin, glutaredoxin and cyclophilin are known to facilitate the regeneration of the reduced (catalytically active) form of peroxyredoxin that plays an important role in reducing ROS formation in plants under biotic and abiotic stress. Generation of ROS, particularly H_2_O_2_, had been proposed to be part of the signaling cascades that lead to protection from stress. Induction of antioxidant enzymes was reported to be a general strategy adopted by plants to overcome oxidative stresses. Peroxidase and CAT function as effective quenchers for ROS [39]. CAT plays an essential role in scavenging from H_2_O_2 _toxicity. The combined action of CAT and POX converts the O_2_^−^ and H_2_O_2_ to water and molecular oxygen (O_2_), thus preventing cellular damage under unfavorable conditions [40]. In the present study potassium plays a key role in the reduction of ROS production by reducing activity of NAD(P)H oxidase and maintaining electron transport [41]. The increase in antioxidant enzyme activity (POX and CAT) was also observed in rice under biotic stress by Liu *et al.* [42] and Liang *et al*. [43]. In the present study, it was observed that POX and CAT had a significant negative correlation with MDA (Table 2), which suggests that the tolerance of rice to water stress effects might be due to increased antioxidant enxyme activity that reduces the production of malondialdehyde. It was reported that production of antioxidant enzymes enabled a decrease in H_2_O_2 _accumulation, eliminating malondialdehyde (MDA), and resulted in cell peroxidation of membrane lipids and maintained cell membrane integrity. This might also serve as a mechanism in ROS scavenging activity of plants under abiotic stress [44]. Table 4 shows that peroxidase and catalase activity contributed high positive direct effects on rice yield compared to malondialdehyde, proline and straw soluble sugar, indicating that one of the tolerance mechanisms of rice under water stress was to enhance production of peroxidase and catalase to prevent cellular damage when potassium were applied to mitigate reduction of rice yield under water stress. This indicates that drought tolerance was increased in 120PW15 treatments by enhancing the production of POX and CAT that are simultaneosly followed by high prodcution of proline and soluble sugar.

**Table 4:** Path coefficient analysis showing direct and indirect effects of biochemical characteristics with rice yield.

Characteristics	Correlation with yield	Direct effect	Indirect effect via					
			POX	CAT	Grains S	Straw S	MDA	Proline
POX	0.7651	0.7114	-	0.0042	0.0301	0.0271	0.0132	0.0014
CAT	0.7762	0.7219	0.0023	-		−0.0071	0.0031	0.0213
Grains S	0.8712	0.7812	−0.0043	0.0041	-	0.0017	0.0543	0.0456
Straw S	0.6521	0.6143	0.0002	0.0015	0.0044	-		0.0213
MDA	0.6543	0.6102	−0.0025	0.0021	0.0001	0.0032	-	
Proline	0.2345	0.1195	0.0032	−0.0043	0.0024	0.0432	0.0321	-

Note: POX = peroxidase; CAT = catalase; Grain S = grain soluble sugar; straw S = straw soluble sugar; MDA = malondialdehyde. Residual factor = 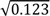 = 0.350.

### 2.3. Total Soluble Sugars

**Figure 2:**
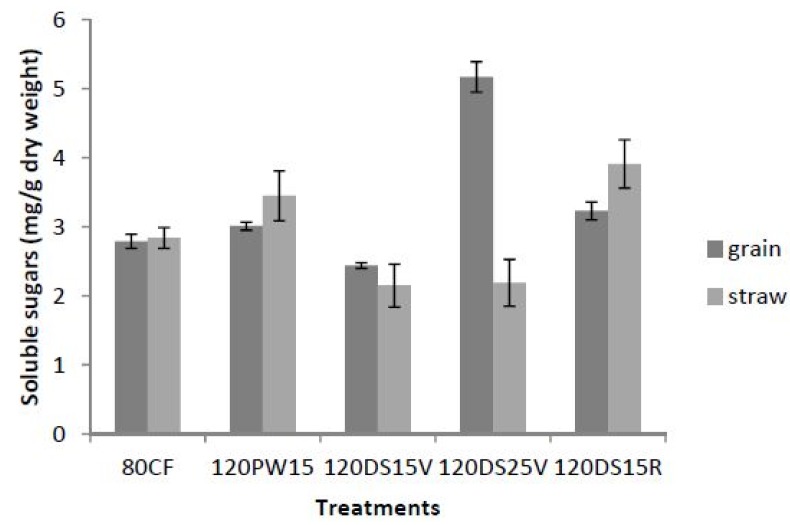
Impact of potassium fertilization on soluble sugars in grain and straw under water stress at different growth stages of rice N = 12. Bars represent standard error of differences between means.

*Notes*: 80CF = continuous flooding + 80 kgK_2_O/ha; 120PW15 = periodical water stress for 15 days + 120 kg K_2_O/ha; 120DS15V = drought stress for 15 days at the vegetative stage + 120 kg K_2_O/ha; 120DS25V = drought stress for 25 days at the vegetative stage + 120 kg K_2_O/ha; 120DS15R = drought stress for 15 days at the reproductive stage + 120 kg K_2_O/ha.

Soluble sugars are influenced by potassium and water stress treatments (*p* ≤ 0.05). It was found that the soluble sugar levels in the grain were higher than in the straw in treatments 120PW15, 120DS15V and 120DS25V (Figure 2). The soluble sugars were balanced in the control (80CF). The high soluble sugars in grains compared to the straw was a good indicator of high translocation of carbohydrates to the sink, and showed that the plants were under recovery from drought stress [45]. Rice yields had a higher correlation coefficent with grain soluble sugars (r^2^ = 0.871; *p* ≤ 0.05; Table 2) than straw soluble sugars (r^2^ = 0.652; *p* ≤ 0.05;Table 2), which signifies that translocation of sugars to grains was very important in producing higher rice yields [46]. This was demonstrated in 120DS25V which achieved a higher rice yield (8.18 tonnes/ha) compared to the other treatments (120PW15, 120DS15V, 120DS15R). A higher straw soluble sugar compared to the grain was an indication of low sink strength and feedback inhibition of photosynthesis [47]. Usually, water stress during the reproductive period induces early senescence, reduces photosynthesis, and shortens the grain-filling period and increases the remobilization of soluble sugars from the grain to the vegetative tissues [44]; this mechanism was justified in 120DS15R, which only produced a yield of 6.09 kg/ha rice.The increase in carbohydrate production by K fertilization under water stress might be due to an increase in starch synthetase (EC 2.4.1.21) activity that enhances the production of carbohydrates [48,49]. Starch synthetase activity is activated by K, and thus with adequate K the levels of starch and soluble sugars will accumulate [50,51,52]. According to Nguyen *et al.* [53], the increased level of sugars might be a natural response in providing protection against oxidant damage by strengthening the antioxidant system in anthers during biotic stress, thus suggesting that the increase in production of soluble sugars might also enhance the tolerance of anthers during water stress, thus increasing the rice yield under biotic stress conditions such as water stress. There was a significant positive correlation between grain soluble sugars and net photosynthesis (r^2^ = 0.923; *p* ≤ 0.05; Table 2) that showed that there was an abundance of soluble sugars in grain rice that corresponded with a higher net photosynthesis and a higher yield in rice. The results showed significant correlations of grain soluble sugars with net photosynthesis (r^2^ = 0.921; *p* ≤ 0.05) and rice yields (r^2^ = 0.871; *p* ≤ 0.05; Table 2;) respectively. In Table 4 it has been shown that grain soluble content was directly involved with rice yield that gave the highest correlation coefficient (0.8712) among the biochemical characteristics, compared to POX (0.7651), CAT (0.7762), Straw soluble sugar (0.6521), MDA (0.6543) and proline (0.2345). The indirect effects have correlation coefficients lower than 0.001. This showed that high yield in rice can be obtained by having high soluble sugar in grain during the grain filling stage. Rice needs a certain time of water stress to enhance remobilization of soluble sugar to the grain during “grain filling” process. In our previous study, drought stress for 25 days initiated during the vegetative stage enhanced grain yield per pot in rice variety MR220. According to Yang *et al.* [54], the induced water stress would increase soluble sugar accumulation to the grain by enhancing sucrose synthase (E.C 2.4.1.13) activity and starch branching enzyme (E.C 2.4.1.18) during the drought period, thus increasing the sink strength to the grain compared to the straw and explained why there were high accumulations of soluble sugar in grain compared to the straw during 25 days of drought stress.

### 2.4. Proline and Lipid Peroxidation

Proline and lipid peroxidation was influenced by the treatments (*p* ≤ 0.05; Table 5). Proline content was found to be highest under 120DS15R (3.44 mg/g fresh weight), followed by 120PW15 (2.75 mg/g fresh weight), 120DS25V (2.73 mg/g fresh weight), 120DS15V (2.01 mg/g fresh weight) and lowest in the control (1.23 mg/g fresh weight). The data indicated that proline content showed significant increases when the rice plants were exposed to cyclic water stress and drought stress, especially at the reproductive stage (120DS25V). The production of proline was enhanced in 120PW15, thus suggesting that the rice built tolerance to water stress by cyclic water stress and potassium fertilization. Interestingly, the production of malondialdehyde was reduced in the control and 120PW15, indicating that the tolerance was built by reducing the malondiadehyde production in plant cells under stress. The increase in proline and reduction in lipid peroxidation was one of the characteristics of rice that can tolerate drought stress, as was observed by Shukla *et al.* [55] and Fu *et al.* [56] in lowland and Dongxiang wild rice, respectively. According to Chutipaijit *et al.* [57], the higher proline accumulation in water-stressed seedlings of tolerant Indica rice genotypes may help to maintain the relative water and MDA levels of rice plants, and lead to preferable growth performance. This indicates that appplication of periodical water stress and potassium fertilizer to rice under water stress mitigates the water stress effects in rice [58,59]. The correlation table showed that MDA and proline had a significant positive correlation (r^2^ = 0.82; *p* ≤ 0.05), and this suggests that the proline and lipid peroxidation mechanism might be related to the water stress defense mechanism in rice [60,61]. The increase in proline and MDA content under abiotic stress was also observed by Wandkhade and Sans [62] and Kong *et al.* [63] in rice exposed to salinity stress. The current results showed that application of potassium during drought or cyclic water availability can mitigate the effects by an increase in the production of proline and MDA in the rice seedlings. In the present study, it was observed that proline and MDA had a significant positive correlation with WUE, which suggests that as rice achieves high WUE with potassium under water stress conditions, the proline and lipid peroxidation content is enhanced. Similar observations were reported by Ahmed *et al*. [64] in barley, where they observed that proline and MDA had a significant possitive correlation with WUE when exposed to drought and salinity stress, suggesting that higher water use efficiency triggered during stress increases the production of proline and lipid peroxidation [65]. In the present study, it was found that proline, POX, CAT and grain and straw soluble sugar had a positive correlation with each other. This suggest as proline accumulated there would be a corresponding accumulation of POX, CAT and soluble sugar in rice subjected to water stress. The same observation was also made by Yang *et al*. [66] and Yan *et al*. [67] in rice exposed to water stress. We also obtained the same result as Sun *et al*. [68], where we observe a weak relationship between MDA with proline, POX and CAT of rice under water stress. Path analysis (Table 4) revealed that most of the biochemical properties (POX, CAT, proline, grain soluble sugar, straw soluble sugar) were involved directly rather than indirectly in the rice yield, thus indicating that these biochemical properties are involved directly in influencing rice yield. In regard to 120PW15 treatments, the increase in production of proline and malondialdehyde might be the indicator of water stress tolerance in MR 220 rice.

**Table 5:** Impact of potassium fertilizationon lipid peroxidation and proline content under water stress at different growth stages of rice. Data are means ± standard error of differences between means (SEM). N = 12. Means not sharing a common single letter were significantly different at *p* ≤ 0.05.

Treatments	MDA (µmol/g fresh weight)	Proline content (mg/g fresh weight)
80CF	11.21 ± 1.21 ^c^	1.23 ± 0.45 ^d^
120PW15	12.32 ± 2.23 ^b^	2.75 ± 0.56 ^b^
120DS15V	12.45 ± 3.12 ^b^	2.01 ± 0.25 ^c^
120DS25V	12.78 ± 1.45 ^b^	2.73 ± 0.08 ^b^
120DS15R	14.21 ± 1.11 ^a^	3.44 ± 0.34 ^a^

*Notes*: 80CF = continuous flooding + 80 kg K_2_O/ha; 120PW15 = periodical water stress for 15 days + 120 kg K_2_O/ha; 120DS15V = drought stress for 15 days at the vegetative stage + 120 kg K_2_O/ha; 120DS25V = drought stress for 25 days at the vegetative stage + 120 kg K_2_O/ha; 120DS15R = drought stress for 15 days at the reproductive stage + 120 kg K_2_O/ha.

### 2.5. Leaf Gas Exchange Properties

The impact of water stress on leaf gas exchange is caused by stomatal closure due to increased ABA production from leaf sources [69]. Water stress in plant-induced stomatal closure depletes intercelullar CO_2_ leading to photoinhibition [70]. Generally, the leaf gas exchange characteristics (net photosynthesis, stomatal conductance, transpiration rate and water use efficiency) were influenced by the treatment effects (*p* ≤ 0.05; Table 4). The net photosynthesis rate was found to be highest in 120DS25V (16.21 µmol m^−2^ s^−1^) followed by 120DS15V (15.41 µmol m^−2^ s^−1^), 120PW15 (15.39 µmol m^−2^ s^−1^), 80CF (15.12 µmol m^−2^ s^−1^) and lowest in 120DS15R (14.17 µmol m^−2^ s^−1^; Table 6). The same trend was observed for stomatal conductance where 120DS25V recorded the highest stomatal conductance (0.3412 mmol/m^2^/s) and the lowest was recorded in 120DS15R (0.310 mmol/m^2^/s). It was observed that the transpiration rate was 4.6% higher than in 80CF. However, transpiration rate was reduced by 7.0, 7.6 and 0.8% in 120PW15, 120DS25V and 120DS15V, respectively, compared to the control (80CF). The highest water use efficiency of 2.17 was obtained in 120PW15. The lowest WUE was recorded on 120DS15R (1.96). There was no significant difference between 120DS15V and 120DS25V in which WUE was recorded as 2.01 and 2.02, respectively. The present results indicated that application of potassium enhanced leaf gas exchange in rice subjected to drought stress until 25 days at the vegetative stage [71,72]. It was also found that potassium fertilization under water stresss conditions at the reproductive stage could minimize drought stress effects such as reduction in net photosynthesis, stomatal conductance and transpiration rate [73]. The result also indicated that the application of cyclic water stress over 15 days with potassium remediation increased water use efficiency. The increase of WUE in the 120PW15 treatment is attributed to an increase in net photosynthesis rate rather than a reduction in transpiration rate, due to the high correlation coefficient for PN (r^2^ = 0.899; *p* ≤ 0.05) compared to E (r^2^ = 0.611; *p* ≤ 0.05; [74]). In path analysis in Table 7, it was showed that enhancement of net photosynthesis and water use efficiency was more important in contributing to rice yield than other leaf gas exchange characteristics. The direct effect for both water use efficiency and net photosynthesis recorded (0.8113 and 0.8021), respectively, were higher than indirect effects. This showed that the stimulation of photosynthesis and increased in the efficiency of rice in using water was a key factor in obtaining higher rice yields. Previous studies have indicated that one strategy of rice subjected to water stress is by enhancing its water use efficiency [75]. A recent study by Yooyongwech *et al*. [45] on Thai Jasmine rice –MT401 has showed that the expression of OsPIP2;1 gene in MT401 rice was enhanced when the plants were exposed to water deficit conditions due to stabilization of WUE at the cellular level. Water use efficiency traditionally defined as the ratio of dry matter produced per unit of water transpired, and constituting one of the key determinants in controlling plant production, it is also referred to as “transpiration efficiency” and estimated from the measures of leaf gas exchange or by using carbon isotope discrimination effects [76,77,78]. In the current study, it was observed that one of adaptation strategies of MR 220 to water stress was to enhance its water use efficiency, especially in 120PW15, thus indicating tolerance to water stress [79]. In the present study, no photoinhibition effect was observed, although the fv/f_o_ and fv/fm decreased from 80CF, 120PW15, 120DS15V, 120DS25V and 120DS15R. This showed that rice variety MR 220 when induced with 120PW15 treatment can be very tolerant to water stress [80].

**Table 6:** Impact of potassium fertilization on the leaf gas exchange under water stress at different growth stages of rice. Data are means ± standard error of differences between means (SEM). N = 12. Means not sharing a common single letter were significantly different at *p* ≤ 0.05.

Treatments	Net photosynthesis (µmol/m^2^/s)	Stomatal conductance (mmol/m^2^/s)	Transpiration rate (mmol/m^2^/s)	Water use efficiency (µmol/m^2^/s CO_2_ assimilated per mmol/m^2^/ water transpired
80CF	15.18 ± 0.24 ^b^	0.33 ± 0.01 ^a^	7.83 ± 0.34 ^b^	1.95 ± 0.03 ^c^
120PW15	15.69 ± 0.45 ^b^	0.35 ± 0.01 ^a^	7.60 ± 0.21 ^b^	2.18 ± 0.02 ^a^
120DS15V	15.72 ± 0.24 ^b^	0.36 ± 0.02 ^a^	7.82 ± 0.13 ^b^	2.01 ± 0.01 ^b^
120DS25V	16.31 ± 0.31 ^a^	0.36 ± 0.01 ^a^	8.09± 0.21 ^a^	2.02 ± 0.02 ^b^
120DS15R	14.28 ± 0.25 ^c^	0.31 ± 0.02 ^a^	7.19 ± 0.12 ^c^	1.98 ± 0.04 ^b^

*Notes*: 80CF = continuous flooding + 80 kg K_2_O/ha; 120PW15 = periodical water stress for 15 days + 120 kg K_2_O/ha; 120DS15V = drought stress for 15 days at the vegetative stage + 120 kg K_2_O/ha; 120DS25V = drought stress for 25 days at the vegetative stage + 120 kgK_2_O/ha; 120DS15R = drought stress for 15 days at the reproductive stage + 120 kg K_2_O/ha]. A = net photosynthesis; g_s_ = stomatal conductance; E = transpiration rate; WUE = water use efficiency; f_o_ = initial fluorescence; f_v_/f_o_ = maximum quantum yield of photochemical and non-photochemical process in photosystem II; f_v_/f_m_ = maximum efficiency of photosystem II and TCC = total chlorophyll. Residual factor = 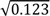 = 0.350.

**Table 7:** Path coefficient analysis showing direct and indirect effects of biochemical characteristics with rice yield.

Characteristics	Correlation with yield	Direct effect	Indirect effect via							
			A	g_s_	E	WUE	f_o_	f_v_/f_o_	f_v_/f_m_	TCC
A	0.8762	0.8113	-	0.0002	0.0331	0.0971	0.0102	0.0311	0.0015	0.0067
gs	0.7762	0.7211	0.0012	-	0.0241	0.0081	0.0011	0.0204	0.0021	0.0054
E	0.6543	0.6065	0.0143	0.0061	-	0.0327	0.0243	0.0562	0.0006	0.0034
WUE	0.8861	0.8021	0.0032	0.0055	0.0014	-		0.0013	0.0012	0.0043
f_o_	0.4562	0.3991	0.005	0.0028	0.0041	0.0012	-	0.0002	0.0234	0.0012
f_v_/f_o_	0.0982	0.0012	0.0032	0.0026	0.0004	0.0131	0.0321	-	0.0042	0.0026
f_v_/f_m_	0.7653	0.7142	0.0024	0.0054	0.0012	0.0091	0.0034	0.1231	-	
TCC	0.8764	0.7921	0.0001	0.0076	0.0123	0.0003	0.0122	0.0062	0.0432	-

*Notes*: A = net photosynthesis; g_s_ = stomatal conductance; E = transpiration rate; WUE = water use efficiency; f_o_ = initial fluorescence; f_v_/f_o_ = maximum quantum yield of photochemical and non-photochemical process in photosystem II; f_v_/f_m_ = maximum efficiency of photosystem II and TCC = total chlorophyll content. Residual factor = 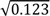
= 0.350.

### 2.6. Cholorophyll Fluorescence Parameters

It was observed that minimal fluorescence (f_o_) value was highest in 120DS15R (711.24), followed by 120DS25V (578.12), 120DS15V (544.21), 120PW15 (523.21) and 80CF(412.23; Table 8). This showed that application of potassium fertilizer during the reproductive stage to mitigate drought stress might be useless due to increases in minimal fluorescence (f_o_) values. The f_o_ is the primary chlorophyll fluorescence yield, that measures the stability of the light harvesting complex [81]. The increase in f_o_ in the present study showed the disruption of photosynthetic apparatus. Previous reports have shown that the increase in f_o_ might be attributed to abiotic stress, particularly water stress [82]. As water stress progressed from 120PW15, 120DS15V, 120DS25V and 120DS15R the f_v_/f_o_ values decreased steadily compared to the control (80CF). The f_v_/f_o_ is an indication of maximum quantum yield of photochemical and non-photochemical processes in photosystem II and correlates with leaf photosynthetic capacity. The reduction in f_v_/f_o_ ratio at the reproductive stage might be due to distruption of photosynthesis in the donor part of photosystem 1 and II. Normally, the f_v_/f_o_ value is in the range of 4–6. However, the range can be different in different plants adapting to different environments [83]. The maximum quantum yield of photosystem II (f_v_/f_m_) also showed the same trends with f_v_/f_o_ ratio. This indicates that f_v_/f_o_ values correlate with f_v_/f_m_ ratio as justified in the correlation table (r^2^ = 0.87; *p* ≤ 0.05; Table 2). The f_v_/f_m_ represent the maximum quantum yield of photosystem II, which is correlated with quantum yield of photosysthesis. It is usually used as an indicator of the photoinhibitor or other injury caused to the photosystem II complex [84]. The values are 0.78–0.84 and almost constant for different plants measured under non-stressed conditions [83,85]. In the present study the f_v_/f_m_ values were less than 0.79 in 120PW15, 120DS15V, 120DS25V and 120DS15R. This suggests that the total amount of light energy transformed in the photosystem II reaction centre was decreased and that rice was stressed under these conditions. This showed that reduction in photochemical activity of PS II can contribute to the limitation in photosynthesis under water stress conditions [84]. It can be concluded that from the drop in f_v_/f_o_ and f_v_/f_m_ and the increase in f_o_ value, the start of water stress can possibly damage the photosynthetic apparatus and lead to disturbances in the photosynthetic process [86]. The results indicate that rice planted under 120PW15 conditions showed resistance to water stress due to the higher f_v_/f_m_ and f_v_/f_o_ and lower f_o_ compared to the control (80CF). The same observation was also noted by Ibrahim *et al*. [87] in *Labisia pumila* varieties where they observed var *alata* and var *pumila* have higher f_v_/f_m_ and f_v_/f_o_ and lower f_o_ andwere more resistance to greenhouse high irradiance compared to var *lanceolata* that was highly light sensitive.

**Table 8:** Impact of potassium fertilization on chlorophyll fluorescence and total chlorophyll content under water stress at different growth stages of rice. Data are means ± standard error of differences between means (SEM). N = 12. Means not sharing a common single letter were significantly different at *p* ≤ 0.05.

Treatments	Minimal fluorescence (f_o)_	Maximum quantum yield of photochemical and non photochemical (f_v_/f_o_)	Maximum quantum yield of photosystem II (f_v_/f_m_)	Total chlorophyll content (SPAD value)
80CF	412.23 ± 22.13 ^d^	3.81 ± 0.67 ^a^	0.79 ± 0.12 ^a^	56.21 ± 4.21 ^a^
120PW15	523.21 ± 12.31 ^b^	3.14 ± 0.87 ^b^	0.72 ± 0.09 ^b^	49.13 ± 3.11 ^b^
120DS15V	544.21 ± 21.23 ^b^	3.23 ± 1.21 ^b^	0.68 ± 0.12 ^c^	35.61 ± 2.76 ^d^
120DS25V	578.12 ± 17.58 ^c^	2.98 ± 0.76 ^c^	0.67 ± 0.14 ^c^	34.21 ± 3.44 ^d^
120DS15R	711.24 ± 25.12 ^a^	2.32 ± 0.81 ^d^	0.59 ± 0.14 ^d^	28.76 ± 2.78 ^c^

*Notes*: 80CF = continuous flooding + 80 kg K_2_O/ha; 120PW15 = periodical water stress for 15 days + 120 kg K_2_O/ha; 120DS15V = drought stress for 15 days at the vegetative stage + 120 kg K_2_O/ha; 120DS25V = drought stress for 25 days at the vegetative stage + 120 kg K_2_O/ha; 120DS15R = drought stress 15 days at the reproductive stage + 120 kg K_2_O/ha].

### 2.7. Relative Chlorophyll Content

The treatments also showed the influence on total cholorophyll content in rice (*p* ≤ 0.05; Table 8). The highest total cholorophyll content was observed in the control treatment (80CF) that recorded 56.1, and this was followed by 120PW15 (49.13), 120DS15V(35.61), and 120DS25V (34.21), and lowest value of 28.76 was recorded in 120DS15R. The data confirmed that the reproductive stage was a very critical period. Potassium fertilization during these period did not enhance total chlorophyll content [88]. Chlorophyll content, which indicates the nitrogen status of the plant, is very important during the grain filling stage. The nitrogen content indicates the sink strength of plants [89], so high chlorophyll content indicates high sink strength of the plants; a higher sink strength would produce higher harvest index and rice yields [23,90]. Usually there is a significant positive correlation between chlrophyll content and rice yield as well as the harvest index [29]. In the present sudy, the correlation table showed that the total cholorophyll content had a significant positive correlation with rice yield (r^2^ = 0.876; *p* ≤ 0.05; Table 2). This indicated that maintaining a higher TCC was important to produce high rice yields under water stress situations. This was reflected in the present study where the control (80CF) which had the highest total chlorophyll content produced the highest rice yield compared to the other treatments (8.65 tonnes/ha). The result was also in agreement with Wankhade and Sanz [61] who observed total chlorophyll content was very important in determining rice yield in *Oryza sativa* L., cv. Taipei 309.

## 3. Experimental

### 3.1. Plant Material, Treatments and Experimental Design

MR220 rice seeds provided by MARDI (Genebank, Seberang Perai, Malaysia) were germinated and transplanted into tanks containing Bakau Series (EC = 2.83 dS m^−1^; pH = 5.1) soil at 15 days after sowing (DAS) in the rainshelter house. Tanks of 55 × 55 × 55 cm size were transplanted with nine seedlings in 3 rows × 3 columns at a spacing of 20 cm. Until 30 DAS seedlings were watered with adequate amounts of water to provide for 1 cm standing water above the soil level. At 30 DAS, the water level in all tanks was raised to 10 cm above the soil level before the first treatment was applied. The experiment was conducted in a randomized complete block design with four replicates. There were five treatments, *i.e.*, 80CF = control flooding + 80 kg K_2_O/ha; 120PW15 = periodical water stress for 15 days + 120 kg K_2_O/ha; 120DS15V = drought stress for 15 days at the vegetative stage + 120 kg K_2_O/ha; 120DS25V = drought stress for 25 days at the vegetative stage + 120 kg K_2_O/ha; 120DS15R = drought stress for 15 days at the reproductive stage + 120 kg K_2_O/ha. The potassium fertilization for every treatment is summarized in Table 9. Other fertilizers including urea (46% N) and triple super phosphate (46% P) were applied at 120 kg N/ha and 70 kg P_2_O_5_/ha, respectively. Standard rice growing procedures were followed throughout the study. Seedling growth and development were monitored daily.

**Table 9:** Potassium fertilization schedule during the experiment.

Treatments	Description	Potassium fertilization schedule (muriate of potash; 60% K_2_O)
80CF	Control flooding + 80 kgK_2_O/ha (Control)	80 kg K_2_O/ha fertilization split into two phases: 30% – 3 leaves stage (15 DAS ^1^) 70% – booting ^2^ (50–55 DAS)
120PW15	Periodical water stress for 15 days + 120 kg K_2_O/ha	120 kg K_2_O/ha fertilization split into three phases: 30% – 3 leaves stage (15 DAS) 30% – booting stage (50–55 DAS) 40% – (80–90 DAS during water stress cycle)
120DS15V	Drought stress 15 days at the vegetative stage + 120 kg K_2_O/ha	120 kg K_2_O/ha fertilization split into two phases: 30% – 3 leaves stage (15 DAS) 70% – booting stage (50–55 DAS)
120DS25V	Drought stress for 25 days at the vegetative stage + 120 kg K_2_O/ha	120 kg K_2_O/ha fertilization split into two phases: 30% – 3 leaves stage (15 DAS) 70% – booting stage (50–55 DAS)
120DS15R	Drought stress for 15 days at the reproductive stage + 120 kg K_2_O/ha	120 kg K_2_O/ha fertilization split into two phases: 30% – 3 leaves stage (15 DAS) 36 kg/ha 70% – booting stage (50–55 DAS)

^1^ DAS = day after sowing; ^2^ booting = bulging of young panicle inside the flag leaf sheath.

### 3.2. Growth and Yield

Plant height was measured from the plant base to the tip of the tallest leaf, and during maturity plant height was measure from the base of the plant to the tip of the tallest flag leaf. Total tillers were counted from three plants per tank on the same day that the plant height data was collected. Meanwhile tiller mortality (TM) was calculated using the formula TM (%) = [(maximum number of tillers − panicles number)/maximum number of tillers × 100]. Rice was harvested when 70% of rice plants showed full ripening colour. The rice panicles (with spikelets) were cut and collected from every tank before being dried in an oven at 60 °C for 2 days. Total panicles produced in each tank were counted. Then the rice spikelets or grains were separated from the panicle and grouped into unfilled grains and filled grains. The 1,000-grain weight and filled spikelets percentage were determined. Meanwhile, the straw biomass was collected from the remaining plants (three hills per tank, without panicles and roots) and dried in an oven at 70 °C for 3 days. The rice grain yield per ha per treatment was computed and the harvest index was calculate based on the ratio of economic yield to total biomass produced. Water productivity in terms of irrigation was determined following the method of Molden *et al*. [91] as yield (kg/ha)/irrigation inflow (L/ha).

### 3.3. Antioxidant Enzyme Activity

#### 3.3.1. Preparation of Enzyme Extracts

To determine the enzymatic activities of the antioxidant proteins, crude enzyme extracts were prepared by homogenizing 500 mg of leaf tissue in extraction buffer containing 0.5% Triton X-100 and 1% polyvinylpyrrolidone in 100 mM potassium phosphate buffer (pH 7.0) using a chilled mortar and pestle. The homogenate was centrifuged at 15,000 rpm for 20 min at 4 °C. The supernatant was used in the enzymatic assays as described below. 

#### 3.3.2. Ascorbate Peroxidase (POX) Activity Assay

Ascorbate peroxidase activity (POX, EC 1.11.1.11) was determined spectophotometrically by the decrease in the absorbance at 265 nm using the method of Nakano and Asada [92]. The reaction mixture contained 50 mM potassium phosphate buffer (pH 7.0), 5 mM ascorbate, 0.5 mM H_2_O_2_ and enzyme extract.

#### 3.3.3. Catalase (CAT) Activity Assay

Catalase activity (CAT; EC 1.11.1.6) was determined by consumption of H_2_O_2 _using the method of Aebi [93]. The reaction mixture (3 mL) contained 50 mM potassium phosphate buffer (pH 7.0), 15 mM H_2_O_2_ and 50 µL enzyme extract. The reaction was initiated by adding the H_2_O_2_. The consumption of H_2_O_2_ was monitored spectrophotometrically at 240 nm for 3 min. Enzyme activity was expressed in micromole per liter H_2_O_2_ min^−1^.

### 3.4. Sucrose Determination

Sucrose was measured spectrophotometrically using the method of Ibrahim and Jaafar [94]. Samples (0.5 g; 0.25 mm) were placed in 15 mL conical tubes, and distilled water added to make up the volume to 10 mL. The mixture was then vortexed and later incubated for 10 min. Anthrone reagent was prepared using anthrone (0.1 g) that was dissolved in 95% sulphuric acid (Fisher Scientific, Omaha, NE, USA, 50 mL). Sucrose was used as a standard stock solution to prepare a standard curve for the quantification of sucrose in the sample. The mixed sample of ground dry sample and distilled water was centrifuged at a speed of 3,400 rpm for 10 min and then filtered to get the supernatant. A sample (4 mL) was mixed with anthrone reagent (8 mL) and then placed ina water-bath set at 100 °C for 5 min before the sample was measured at an absorbance of 620 nm using a spectrophotometer (Model UV160U; Shimadzu Scientific, Kyoto, Japan). The soluble sugar in the sample was expressed as mg sucrose g^−1^ dry sample.

### 3.5. Malondialdehyde (MDA) Content

Lipid peroxidation of plant parts was estimated by the level of malondialdehyde (MDA) production using the thiobarbituric acid (TBA) method as described by Ibrahim and Jaafar [93]. One gram of ground (0.25 mm) plant sample was homogenized in a mortar and pestle with 0.5% trichloracetic acid (TCA, 1 mL). The homogenate was centrifuged at 9,000 rpm for 20 min. The supernatant (0.5 mL) was mixed with 20% TCA (2.5 mL) containing 0.5% TBA and heated in a boiling water bath for 30 min and allowed to cool in anice bath quickly. The supernatant was centrifuged at 9,000 rpm for 10 min, and resulting supernatant was used for determination of MDA content. Absorbance at 532 nm was recorded.

### 3.6. Proline Determination

About 5 mg of lyophilized and homogenized samples were extracted in 0.5 mL of 3% 5-sulpho-salicylic acid for 15 min. The samples were then centrifuged at 21,000 *×g* for 15 min. The clear supernatant (200 µL) was transferred to polypropylene screw cap vials, and 200 µL of concentrated formic acid and 400 µL of 3% ninhydrin reagent in 2-methoxyethanol were added. Samples were heated for 0.5 h at 100 °C in a water-bath, and then transferred to 96-well plates. Absorbance was measured at 514 nm on a micro-plate reader (Synergy 2, Bio-Tek, Winooski, VT, USA). The level of proline was expressed in µg g^−1^ DW (dry weight of leaves).

### 3.7. Leaf Gas Exchange

The measurement was obtained using a closed infra-red gas analyzer LICOR 6400 Portable Photosynthesis System (IRGA, Licor Inc., Lincoln, NE, USA). The measurements used optimal conditions set at 400 µmol mol^−1^ CO2, 30 °C cuvette temperature, 60% relative humidity with air flow rate set at 500 cm^3^ min^−1^, and modified cuvette conditions of 225, 500, 625 and 900 µmol/m^2^ photosynthetically photon flux density (PPFD), respectively. Gas exchange measurements were carried out between 09:00 to 11:00 a.m., using fully expanded young leaves numbered 3 and 4 from plant apex to record net photosynthesis rate (A). The operation was automatic and the data were stored in the LI-6400 console and analyzed by “Photosyn Assistant” software (version 1.0; Dundee Scientific: Dundee, Scotland, UK, 2000). Several precautions were taken to avoid errors during measurements. Leaf surfaces were cleaned and dried using tissue paper before being enclosed in the leaf cuvette. Data of net photosynthesis rate (µmol m^−2^ s^−1^), stomata conductance (μmol H_2_O m^−2^ s^−1^), transpiration rate (mmol m^−2^ s^−1^), and IWUE (mol/m^2^/s water transpired/mmol/m^2^/CO_2_ assimilated) was collected during the measurement [95].

### 3.8. Chlorophyll Fluorescence

Measurements of chlorophyll fluorescence were taken from fully expanded second leaves. Leaves were darkened for 15 min by attaching light-exclusion clips to the central region of the leaf surface. Chlorophyll fluorescence was measured using a portable chlorophyll fluorescence meter (Handy PEA, Hansatech Instruments Ltd., Norwich, UK). Measurements were taken at >3,000 µmol/m^2^/s and recorded for up to 5 seconds. The fluorescence responses were induced by emitting diodes. Measurement of f_o_ (initial fluorescence), f_m_ (maximum fluorescence) and f_v_ (variable fluorescence) were obtained from this procedure. f_v_ is derived as the difference between f_m_ and f_o_. Fluorescence values recorded, include the initial/minimal fluorescence (f_o_), the ratio of variable to maximum fluorescence (f_v_/f_m_) which represents the maximum quantum yield of photosystem II (PS II), and the ratio of variable to minimum fluorescence (f_v_/f_o_) which estimates the maximum primary yield of photochemistry of PS II. The f_m_ is the maximal fluorescence value, and f_v_ is the variable fluorescence calculated as f_m_ − f_o_ [96].

### 3.9. Chlorophyll Measurement

The measurement of total chlorophyll content was determined by using SPAD meter 502 (Minolta Inc; Lincoln, NE, USA).

### 3.10. Statistical Analysis

Data were analyzed using the analysis of variance procedure in SAS version 17. Means separation tests between treatments was performed using Duncan multiple range test (DMRT) and standard error of differences between means were calculated with the assumption that data were normally distributed and equally replicated. Correlation and path coefficient analysis were computed by following the standard statistical procedure by Anwarmalik [97].

## 4. Conclusions

The imposition of periodical water stress over 15 days and potassium (120PW15) fertilization have been shown to enhanced rice tolerance to water stress. Although rice yield is slightly higher in 120DS25V the application of 120CW15 was more water efficient. This has the possibility to be applied by rice growers in Malaysia under limited water sources. Increase in drought tolerance in 120CW15 was shown by increased water use efficiency, POX, CAT, proline, f_v_/f_m_, and lower f_o_. Path coefficient analysis revealed that most of parameters contribute directly rather than indirectly to rice yield. In this experiment, there were four factors that that directly involved with rice yield: *i.e*., grain soluble sugar, photosynthesis, water use efficiency and total chlorophyll content. The residual factors to rice yield are observed to be quite low in the experiment (0.350), indicating that rice yield was mainly influenced by the parameters measured during the study.
